# Public Perceptions Regarding Use of Virtual Reality in Health Care: A Social Media Content Analysis Using Facebook

**DOI:** 10.2196/jmir.7467

**Published:** 2017-12-19

**Authors:** Michelle Sophie Keller, Hannah J Park, Maria Elena Cunningham, Joshua Eleazar Fouladian, Michelle Chen, Brennan Mason Ross Spiegel

**Affiliations:** ^1^ Cedars-Sinai Center for Outcomes Research and Education Cedars-Sinai Medical Center Los Angeles, CA United States; ^2^ Division of Informatics Department of Biomedical Sciences Cedars-Sinai Medical Center Los Angeles, CA United States; ^3^ Department of Health Policy and Management University of California, Los Angeles Los Angeles, CA United States

**Keywords:** social media, virtual reality, qualitative research

## Abstract

**Background:**

Virtual reality (VR) technology provides an immersive environment that enables users to have modified experiences of reality. VR is increasingly used to manage patients with pain, disability, obesity, neurologic dysfunction, anxiety, and depression. However, public opinion regarding the use of VR in health care has not been explored. Understanding public opinion of VR is critical to ensuring effective implementation of this emerging technology.

**Objective:**

This study aimed to examine public opinion about health care VR using social listening, a method that allows for the exploration of unfiltered views of topics discussed on social media and online forums.

**Methods:**

In March 2016, NBC News produced a video depicting the use of VR for patient care. The video was repackaged by NowThis, a social media news website, and distributed on Facebook by Upworthy, a news aggregator, yielding 4.3 million views and 2401 comments. We used Microsoft Excel Power Query and ATLAS.ti software (version 7.5, Scientific Software Development) to analyze the comments using content analysis and categorized the comments around first-, second-, and third-order concepts. We determined self-identified gender from the user’s Facebook page and performed sentiment analysis of the language to analyze whether the perception of VR differed by gender using a Pearson’s chi-square test.

**Results:**

Out of the 1614 analyzable comments, 1021 (63.26%) were attributed to female Facebook users, 572 (35.44%) to male users, and 21 (1.30%) to users of unknown gender. There were 1197 comments coded as expressing a positive perception about VR (74.16%), 251 coded as expressing a negative perception and/or concern (15.56%), and 560 coded as neutral (34.70%). Informants identified 20 use cases for VR in health care, including the use of VR for pain and stress reduction; bed-bound individuals; women during labor; and patients undergoing chemotherapy, dialysis, radiation, or imaging procedures. Negative comments expressed concerns about radiation, infection risk, motion sickness, and the ubiquity of and overall dependence on technology. There was a statistically significant association between the language valence of the Facebook post and the gender of the Facebook user; men were more likely to post negative perceptions about the use of VR for health care, whereas women were more likely to post positive perceptions (*P*<.001).

**Conclusions:**

Most informants expressed positive perceptions about the use of VR in a wide range of health care settings. However, many expressed concerns that should be acknowledged and addressed as health care VR continues to evolve. Our results provide guidance in determining where further research on the use of VR in patient care is needed, and offer a formal opportunity for public opinion to shape the VR research agenda.

## Introduction

### Virtual Reality in Health Care Settings

Virtual reality (VR) technology provides an immersive environment that enables users to have modified experiences of reality [1,2]. To date, VR has been used in various health care settings to help treat anxiety disorders, reduce fall risks in older patients, control pain, manage obesity, support physical rehabilitation, and distract patients during wound care [1-9]. By stimulating the visual and proprioceptive senses, VR acts as a distraction to limit the processing of nociceptive stimuli while refocusing the brain on cognitively stimulating, positive, and potentially therapeutic experiences [2]. In a meta-analysis of randomized controlled trials, we found that VR is generally effective and well tolerated by patients across a range of clinical settings, although the existing literature is hampered by small studies of varying quality [10].

Despite increasing awareness about VR and its potential benefits, it remains unclear whether and how best to scale this technology in clinical practice. There are also questions about whether some patients are willing to accept VR in the clinical settings. We previously assessed the acceptability of VR in hospitalized patients [11] and found that most patients find VR to be a positive and pleasant experience that eases anxiety and provides an escape from the confines of a distressful illness experience. Most patients report a willingness to use VR again if given the opportunity. However, we also found that younger patients are more willing to use VR than older patients; that some patients find the technology uncomfortable, intrusive, or confusing to use; and that patients occasionally report that the headsets are difficult to operate, can induce vertigo, or are of unclear benefit. In short, this study suggests that introducing VR into clinical practice requires careful thought, consideration of patient preferences, and an understanding of the risks and benefits of this emerging technology.

The potential health care uses of VR are far-reaching, but acceptability of the technology is an important consideration toward enabling its successful implementation. Addressing concerns about the safety, effectiveness, usability, and accessibility of VR is critical to ensuring that VR interventions and programs are effective. Moreover, it is important to survey public perception not only about concerns surrounding VR but also about use cases in which VR may be most impactful. Patients should have a voice in determining whether, when, and where to implement VR in their own care.

### Using Social Listening to Examine Opinions of Virtual Reality

Few studies have examined user perceptions of VR, and of these studies, most focused on narrow applications of VR in clinical settings [12-15]. In this study, we used social listening techniques to examine unsolicited comments posted on Facebook in response to an online video about the use of VR in health care. We selected to study the Facebook posts in response to the video given that people use the immensely popular social network to share opinions about various topics with their online communities. Facebook is the largest social network with respect to logged-in users; in June 2017, the site had 2 billion monthly active users [16]. In addition, Facebook was the ideal platform to study public opinions about VR, as the video was posted on the site by an online news aggregator (Upworthy) that specializes in posting viral videos. This presented a natural opportunity to study the Facebook posts about VR without explicitly soliciting opinions from users.

The objective of this study was to use social listening methods to examine how individuals perceive health care VR, including understanding general sentiments about the technology, concerns about the use of health care VR, and which settings may be useful future areas for VR research. The results can elucidate facilitators and barriers to the dissemination and implementation of VR in health care. We use quantitative content analysis to characterize online opinions about the use of VR for patient care.

## Methods

### Study Overview

In March 2016, NBC News produced a video depicting the use of VR for patient care based on our research at Cedars-Sinai Medical Center and other sites employing VR in clinical practice. The video was edited and repackaged by “NowThis,” a digital news company that distributes content to social media sites, and posted on Facebook by Upworthy, yielding 4.3 million views, 36,000 shares, 67,000 “likes,” and 2401 spontaneous comments as of December 2016. The video depicts hospitalized patients using VR headsets, features children using VR to ease the experience of cancer treatment, includes brief interview quotes about how VR can be used in health care, and references the emerging role of VR for managing depression, anxiety, and various types of pain [17].

In this study, we expand on our previous research [11] by examining the Facebook posts submitted in response to the video. We employ social listening techniques to capture online public opinion. Social listening offers unique advantages compared with traditional survey methods. One of these advantages is the ability to capture unsolicited discussions among participants without a researcher present, thus overcoming the Hawthorne effect, where individuals may change their behavior when they know they are being studied [18]. Social listening can catalogue opinions from large informant groups and from those who might not otherwise participate in a research study. Furthermore, social listening allows researchers to capture data from a wide demographic and geographic spectrum.

Social listening is an established and efficient way to study online communities [19]. Previous studies have used Facebook to examine the social media activity of clinicians and pharmacists [20,21]; to evaluate Facebook groups focused on diabetes and explore the information that patients request, the unsolicited information that is provided, and the nature of the virtual communities that congregate on Facebook [22]; and to examine risk perceptions of obesity among social media users [23]. We build on this methodology by using responses to the VR video posted on Facebook as a virtual focus group to characterize public knowledge, attitudes, beliefs, and preferences about the use of VR in health care. We used qualitative methods to develop a list of themes in the data and used this list to apply the codes to the rest of the Facebook posts. We subsequently quantified the results for analysis.

### Gender and Social Media Use

We examined the gender of the informant, given that previous studies have found that men and women express themselves in different ways on social media. Several studies examining interactions and posts on Facebook found that there are notable variations in the use of language on social media by gender [24,25]. Men are more likely to use swear words, whereas women are more likely to use positive, emotion-related words such as “excited” [24,25]. Other studies have found that women are more likely to discuss social relationships (eg, friendships and family), whereas men are more likely to discuss topics such as online gaming, sports, and political topics [25]. Additionally, given that various demographic characteristics, including gender, have been found to be important factors in the adoption and diffusion of technology [26-28], we wanted to explore whether the same holds true for the use of VR. Previous studies have found, for instance, that men value the relative advantage and overall usefulness of technology more highly, whereas women tend to value ease of use [27,28].

### Data Collection and Analysis

We used Microsoft Excel Power Query (2016, Microsoft Corporation) to extract 2401 Facebook comments written in response to the video posted as of 4:20 pm on March 7, 2016. Out of the 2401 comments, we analyzed 67.22% (n=1614) of posts that expressed a measurable sentiment and excluded posts in which users simply tagged friends or included uninterpretable symbols with no other information.

We used the qualitative analysis software ATLAS.ti (Scientific Software Development, Berlin, Germany) to code the Facebook posts [29]. We used ATLAS.ti, given the functionality of the software to support multiple coders and to perform quantitative analyses based on the codes and categories. We used multiple coders (HJP, MEC, and JEF) to enhance the coding process; using multiple coders allowed for the inclusion of multiple perspectives during the code and category development and the ability to discuss coding disagreements among the group. The first round of inductive coding was used to generate a codebook of themes grounded in the data. We used a consensus process to agree on the final list of codes. The coders then iteratively coded the data several times to categorize each of the Facebook posts into the sentiment categories (positive, negative, and neutral) and major (eg, mental health uses and pain relief) and minor (eg, VR as helpful for anxiety, depression, or stress) themes.

Facebook posts could contain more than one theme if they expressed multiple messages or sentiments within the same post. The unit of analysis was the entire Facebook post. We subsequently used ATLAS.ti to generate code count histograms within major and minor themes. We used the sentiment values and major and minor themes to create a map of attitudes, beliefs, and preferences about the use of VR in health care. We compared perceptions and beliefs by informant gender—a demographic variable accessible through each informant’s Facebook page. If self-identified gender was missing, then we coded that individual’s gender as unknown.

We used the ATLAS.ti code cooccurrence tool to explore patterns among code frequencies regarding gender differences in the categories of Facebook posts, as have been reported in previous research [30]. We used Pearson chi-square tests to examine the association between gender and sentiment valence of Facebook post.

The study was reviewed and approved by the Cedars-Sinai Medical Center's Institutional Review Board (Pro00044905). No individual subjects were contacted. The only study data used were public posts on Facebook, accessed in full accordance with the Facebook privacy policy.

## Results

### Facebook Comments by Gender, Sentiment, and Theme

Out of the 1614 Facebook comments analyzed, 1021 (63.25%) were attributed to female Facebook users, 572 (35.43%) to male users, and 21 (1.30%) to users of unknown gender (Table 1). Overall, 1197 (74.16%) comments were coded as expressing a positive perception about VR, 251 (15.55%) coded as a negative perception or concern, and 560 (34.70%) coded as neutral. Comments often expressed overlapping themes; thus, the percentage total does not equal 100%. Thematic analysis of the of the comments yielded 50 unique codes, including 27 positive perception codes, 18 negative perception codes, and 5 neutral codes.

**Table 1:** Sentiment type of Facebook comments by gender.

Gender of Facebook user	Number of codes by language valence		
	Positive	Negative	Neutral
Female	873	100	378
Male	317	148	250
Total codes^a^	1190	248	628

^a^Each comment may include multiple statements; therefore, the number of codes is greater than the number of overall comments.

Multimedia Appendix 1 depicts the thematic network encompassing positive, negative, and neutral sentiments. The network indicates that opinion about the role of VR in health care is varied; informants reported diverse attitudes, beliefs, preferences, and concerns about the use of the technology. Multimedia Appendix 2 shows specific examples of Facebook comments left in response to the video.

### Positive Perception Network

Table 2 lists the 27 codes for positive beliefs, attitudes, and preferences toward VR. Positive perceptions were categorized into the following 2 major groups of first-order concepts: (1) *specific health care uses* and (2) *general uses*. The category *specific health care uses* included the following 5 second-order concepts organized around the use of VR in various settings and conditions: (1) *lack of mobility*, (2) *pain*, (3) *mental health*, (4) *treatment and rehabilitation*, and (5) *drugs*. Secondary concepts under *general uses* included *interest in VR technology* and *desire for personal use and to share with others.*

The second-order concepts of both primary groups encompass additional third-level concepts specifying positive uses and reactions to health care VR. For example, informants identified both acute and chronic pain conditions that may benefit from VR.

Under *specific health care uses,* the most common grouping of Facebook posts pertained to using VR to combat a lack of mobility (n=153), followed by managing pain (n=42), and the use of VR to positively influence mental health or for use in mental health treatment (n=31). Informants expressed opinions about how VR could benefit a variety of populations with a lack of mobility, including patients with long-term hospital stays (n=30), elderly patients who cannot move or travel (n=25), children in the hospital (n=15), cancer patients undergoing chemotherapy or radiation treatment (n=9), patients receiving scans or undergoing other types of imaging (n=8), and wheelchair-bound individuals (n=3). In addition, informants believed that VR could be beneficial for populations facing a lack of mobility, distracting individuals from boredom, and encouraging mental stimulation (n=60), as well as encouraging movement (n=3). Specifically, for mental health, informants noted how VR could be used to manage stress (n=14), anxiety (n=10), and depression (n=7).

General positive responses include the following second-order groups: (1) *interest in VR technology* and (2) *desire for personal use and to share with others*. *Interest in VR technology* had third-order concepts of strong general interest (n=665), positive use of technology (n=80), and remarks expressing that VR was better than television or movies (n=9). The most common third-order concept was *general interest in VR* (n=665), principally comprising nonspecific positive reactions to VR in health care (eg, “cool,” “awesome,” and “I love this!”). Secondary concepts under *desire for personal use and to share with others* include individuals expressing interest in VR for their personal use (n=61) or interest in trying VR (N=49), and wishing that VR had been available in their previous hospital stay (n=39) or that the technology was available or had been available for friends and family members in the hospital (n=31).

Acute pain conditions (quaternary concepts) under the second-level concept *pain* and under the third-level concept *acute pain* identified by informants include VR benefits for patients in labor and delivery (n=15), dentistry (n=13), burns (n=2), and dialysis (n=1) settings.

### Negative Perception Network

Table 3 lists the 18 codes for negative beliefs, attitudes, and preferences toward VR. Negative perceptions were categorized into the following 2 major groups of first-order concepts: (1) *concerns of VR effects in health care* and (2) *general hesitation/negative reaction*. *Concerns of VR effects in health care* include the following 3 second-order concepts: (1) *paranoia or barriers*, (2) *threats to patient health*, and (3) *negative effects on specific groups*. *General hesitation/negative reaction* includes 2 second-order concepts, including *disinterest* and *more research necessary to support claims*.

The second-order concepts mapped to several third-order concepts specifying the negative reactions and concerns elicited by informants. For example, third-order groups under *paranoia or barriers* revealed user concerns over insurance coverage of VR and costs of use (n=77), VR increasing the societal dependence on technology (n=26), concerns about the information that is transmitted to the user (n=7), VR as a barrier to discharge (n=3), VR creating difficulties in caring for patients (n=2), and concerns about what happens in the room while the patient uses VR (n=2).

The most common codes under *concerns of VR effects in health care* included paranoia or barriers concerning the use of VR (n=153), followed by VR as a threat to patient health (n=31) and negative effects of VR on specific groups (n=16). Informants identified 5 potential threats to patient health with the use of VR in health care, including motion sickness (n=11), vision complications (n=10), infections due to bacteria and other microorganisms because of sharing equipment (n=4), the potential for radiation from the mobile phone to contribute to cancer (n=3), and concerns about patients falling off the bed when using VR (n=3). Informants expressed concerns about pregnant patients and patients in labor (n=16), psychiatric or traumatic brain injury patients (n=3), and those suffering from mental illness (n=3).

Other users wrote about various general hesitations and reactions, including VR as being generally unnecessary (n=42), the ability for drugs and alcohol to achieve the same goal (n=25), the belief that the idea can be improved or advanced (n=17), and the need for more research to support the claims (n=3).

**Table 2:** Distribution of positive beliefs, attitudes, and perceptions toward virtual reality in Facebook comments posted in response to the video.

Positive theme of Facebook comment identified through qualitative analysis			Value (N=1197^b^), n (%)
**General uses (N=934)**			
	**Interest in VR^a^technology (N=754)**		
		General interest	665 (55.56)
		Positive use of technology	80 (6.68)
		Better than TV or movies	9 (0.75)
	**Desire for personal use and to share with others (N=180)**		
		General personal use	61 (5.09)
		Interest in trying VR	49 (4.09)
		Wishes that they had VR in previous hospital stay	39 (3.26)
		Wishes that they had VR for friend and family members in hospital	31 (2.59)
**Specific health care uses (N=263)**			
	**Lack of mobility (N=153)**		
		Distract from boredom, encourage mental stimulation	60 (5.01)
		Long-term hospital stays	30 (2.51)
		Elderly patients who cannot move or travel	25 (2.09)
		Children in hospital	15 (1.25)
		Cancer patients undergoing chemotherapy or radiation	9 (0.75)
		Patients undergoing scans/imaging	8 (0.67)
		People who are wheelchair-bound	3 (0.25)
		Encourage movement	3 (0.25)
	**Pain (N=42)**		
		Labor delivery (acute)	15 (1.25)
		Dentistry (acute)	13 (1.09)
		Chronic pain (chronic)	11 (0.92)
		Burns (acute)	2 (0.17)
		Dialysis (acute)	1 (0.08)
	**Mental health (N=31)**		
		Stress	14 (1.17)
		Anxiety	10 (0.84)
		Depression	7 (0.58)
	**Treatment and rehabilitation (N=19)**		
		General rehabilitation	13 (1.09)
		Adjunct to other therapies	6 (0.50)
	**Drugs (N=18)**		
		Adjunct to other drugs	11 (0.92)
		Reduces the need for drugs	7 (0.58)

^a^VR: virtual reality.

^b^Total does not add up to 100% due to multiple codes per Facebook comment and rounding.

**Table 3:** Distribution of negative beliefs, attitudes, and perceptions toward virtual reality in Facebook comments posted in response to the video.

Negative theme of Facebook comment identified through qualitative analysis			Value (N=251^a^), n (%)
**Concerns of VR^b^effects in health care (N=164)**			
	**Paranoia or barriers (N=117)**		
		Insurance coverage and cost	77 (30.7)
		Societal dependence on technology	26 (10.7)
		Information that is transmitted to user	7 (2.8)
		Barrier to discharge	3 (1.2)
		Creates difficulties in caring for patient	2 (0.8)
		What happens in the room while patient uses VR	2 (0.8)
	**Threats to patient health (N=31)**		
		Motion sickness	11 (4.4)
		Vision complications	10 (4.0)
		Spread of bacteria	4 (1.6)
		Radiation/emissions/cancer	3 (1.2)
		Falling off bed	3 (1.2)
	**Negative effects on specific groups (N=16)**		
		Pregnancy or labor	10 (1.6)
		Psychiatric or brain injury	3 (1.2)
		Mental illness	3 (1.2)
**General hesitation/negative reaction (N=87)**			
	**Disinterest (N=67)**		
		Generally unnecessary or excessive	42 (16.7)
		Drugs and alcohol achieve the same goal	25 (9.7)
	**More research necessary (N=20)**		
		Idea can be improved or advanced	17 (6.78)
		More research needed to support claims	3 (1.2)

^a^Total does not add up to 100% due to multiple codes per Facebook comment and rounding.

^b^VR: virtual reality.

**Figure 1:**
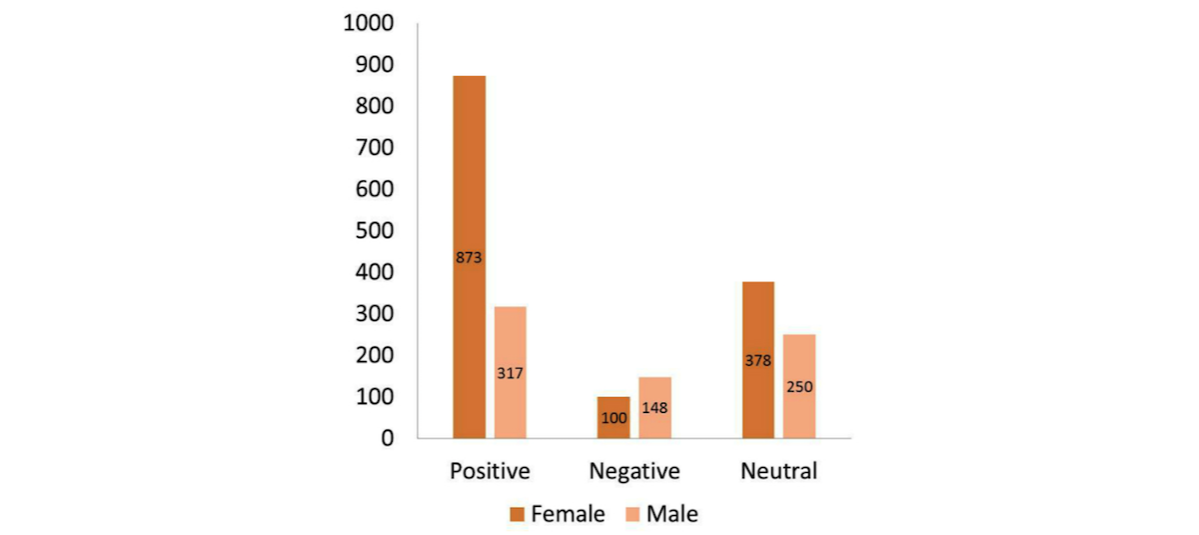
Distribution of Facebook statements regarding virtual reality technology in health care by gender.

### Neutral Perception Network

There were 5 codes for neutral beliefs, attitudes, and preferences toward VR. Neutral responses were grouped into either *curious/inquiring* responses or *prior exposure/knowledge* responses. *Curious/inquiring* responses were grouped into the following second-order responses: (1) *wants to share information*, (2) *long-term outcomes/future uses of VR*, and (3) *availability outside of the United States*. Responses in the first-order group *prior exposure/knowledge* comprised users who *have already heard about this technology* and users who *compare VR to an existing project or media*. The most common second-order concept was *wants to share information* (n=349). This was followed by the code *compares VR to an existing project or media* (n=140).

### Type of Comments by Gender

Pearson’s chi-square test was used to determine if there was an association between language valence (positive, negative, or neutral) and gender. Men were significantly more likely to exhibit negative perceptions about the use of VR in health care than women (*P*<.001; Figure 1).

## Discussion

### Principal Findings

This case study provides rich data to better understand how the public perceives the use of VR in a health care setting. The results provide innovative, crowdsourced ideas for how to shape VR implementation in health care. For example, the study informants noted that VR could be used in areas such as labor and delivery, dentistry, chemotherapy administration, and in patients undergoing imaging tests. There is preliminary research in some of these areas [31-33], and our findings illustrate that there is public interest in using health care VR for these types of applications. Health care organizations and the VR research community may use the resulting thematic network (Multimedia Appendix 1) to help determine where further research on the use of VR in patient care is needed.

A 2012 review examining users’ perceptions of VR game-based interventions in the rehabilitation setting noted that users were primarily concerned with the technological limitations of VR, the ability of the user to use the VR system independently, the desire to engage in novel physical and cognitive challenges using VR, the ability to connect with other users using VR during rehabilitation, the ability to receive feedback on progress during rehabilitation sessions, and a desire for rich and varied virtual environments [34]. Interestingly, this study found that although the study informants in our study noted that VR could be used for rehabilitation and to encourage movement, their concerns were quite different from individuals who had used VR in the rehabilitation setting. Although study informants in our study focused on cost, potential technology dependence, and safety issues (eg, falling off the bed), users who had used VR in the health care setting noted different types of concerns, including the desire to use VR in a more social manner, the failure of the technology to meet expectations, the ability to use the technology without assistance, and the desire for more realistic virtual worlds. Actual users of VR were less concerned with overdependence on technology, even those who had used game-based therapies, illustrating that clinicians hoping to use VR may need to address these unfounded fears with new users. This demonstrates that although our findings may be useful in facilitating the dissemination of VR by reducing barriers to use for novice users, more qualitative research is needed to understand how to improve the technology so that it can be used in specific health care settings.

Our findings from this natural experiment indicate that online perceptions of health care VR is generally positive, and most informants believe the technology can benefit various populations in diverse clinical settings. Informants expressed interest in using VR for patients with diminished mobility (eg, those experiencing long-term hospital stays; frail individuals; those receiving chemotherapy, dialysis, radiation, or imaging; and wheelchair-bound individuals) as a drug-free treatment for acute or chronic pain, and for those struggling with mental health conditions such as anxiety and depression. Our findings are in line with those of other studies, which have found that individuals perceive VR as a form of therapy with benefits for the mind and body [35,36]. Previous studies have found VR to be effective in reducing chronic pain [37], aiding in stroke rehabilitation [14,35], improving movement in Parkinson's patients [38-40], treating nicotine addiction [41], reducing anxiety and post-traumatic stress disorder [42], and aiding in the rehabilitation of traumatic brain injury [43].

### Concerns About Virtual Reality and Implications for Use

However, it is important to acknowledge that many informants in this study expressed concerns about using VR for patient care. There are concerns about costs, whether insurance will cover VR as a therapeutic option, and an overarching concern about increasing dependence on technology. The rising cost of health care is a critical issue overall, and previous research has found that individuals are concerned about the gap between those who can afford experimental treatment and those who struggle to get basic medical care [43]. The results from this study illustrate that apprehension about access to and affordability of new technologies is of foremost importance in the public eye. The adoption and dissemination of VR in the health care setting, therefore, will be dependent on how health care systems price this service and whether insurance companies choose to cover the technology and associated VR services. Future studies that examine the cost-effectiveness of adding VR to standard inpatient care will be useful in understanding the added value of VR.

Interestingly, another important barrier to use expressed by informants was the potential of VR to add to the overdependence of technology in society. This finding reveals that individuals feel some trepidation about the current use—and overuse—of technology in society, and that they may not be willing to continue to accept more forms of technology in settings such as health care, despite the potential clinical benefits. Although some individuals may see the use of VR as a way to escape a hospital room, others may be hesitant to use and engage with additional consumer-facing technologies that could prove to be addictive. Researchers should examine whether game-based VR applications, such as those used in rehabilitation settings, have the potential to be addictive. If users are assured that these applications can be used without fears of overuse, they may be more likely to be open to using the technology.

### Gender Differences in Virtual Reality Use

We found that female users are more likely to comment positively, whereas male users express more concerns about VR for patient care. Our findings are in line with previous research showing that women express positive sentiments more frequently than men in a social media context [25]. We also found that women are more likely to post any opinion about VR, independent of sentiment, which is also consistent with previous research about gender use and social media. Men have been found to be less likely to engage in social media and less likely to use Facebook [44,45]. Women have also been found to express themselves in warmer, more compassionate, and more polite tones than men on social media sites [24].

This study also aligns with other research that finds gender differences in attitudes and adoption of technology [27], including the acceptance and use of technologies such as fitness trackers [46], mobile chat services [47], video games [48], and Internet use overall [49]. Thus, VR, much like other consumer-facing technologies, may be adopted and used by diverse groups in different manners. Researchers studying the diffusion of VR technology should incorporate these gender differences into models of diffusion and should also explore more in depth how different demographic characteristics influence the use and acceptance of VR.

This study has important limitations. First, the video published by Upworthy portrayed VR in a generally positive light, which could bias Facebook users toward commenting more positively. However, our results indicate that the video still prompted a wide range of far-reaching negative comments, indicating a diversity of sentiment. Second, our sample was limited to people who have a Facebook account and who viewed the video on their feed; similar to other surveys, our sample may not express sentiments that represent the larger population. Compared with the general population, informants in this study may be more interested in new technologies or have social networks that view new technologies in a more favorable light. Third, social listening can only capture comments from users who choose to post. Thus, individuals who prefer to view content but do not post are not represented in our analysis. Another limitation is the relatively narrow scope of the study: we focused on responses to one video posted on Facebook. Future research may expand on this study by exploring public opinion about VR based on various sources. Finally, we were limited in the type of demographic data that we could capture through Facebook.

In conclusion, this social listening analysis of a large social media sample indicates wide acceptance of and interest in using VR for patient care but also reveals concerns that should be acknowledged and addressed as health care VR continues to evolve.

## Appendix

Multimedia Appendix 1 Thematic network of sentiment in Facebook comments posted in response to virtual reality (VR) video.

Multimedia Appendix 2 Examples of Facebook comments about virtual reality (VR) technology in the health care setting.

## Abbreviations

VR: virtual reality

## References

[1] Malloy KM, Milling LS (2010). The effectiveness of virtual reality distraction for pain reduction: a systematic review. Clin Psychol Rev.

[2] Li A, Montaño Z, Chen VJ, Gold JI (2011). Virtual reality and pain management: current trends and future directions. Pain Manag.

[3] Carrougher GJ, Hoffman HG, Nakamura D, Lezotte D, Soltani M, Leahy L, Engrav LH, Patterson DR (2009). The effect of virtual reality on pain and range of motion in adults with burn injuries. J Burn Care Res.

[4] Furman E, Jasinevicius TR, Bissada NF, Victoroff KZ, Skillicorn R, Buchner M (2009). Virtual reality distraction for pain control during periodontal scaling and root planing procedures. J Am Dent Assoc.

[5] Garrett B, Taverner T, Masinde W, Gromala D, Shaw C, Negraeff M (2014). A rapid evidence assessment of immersive virtual reality as an adjunct therapy in acute pain management in clinical practice. Clin J Pain.

[6] Gold JI, Kim SH, Kant AJ, Joseph MH, Rizzo AS (2006). Effectiveness of virtual reality for pediatric pain distraction during i.v. placement. Cyberpsychol Behav.

[7] Hoffman HG, Doctor JN, Patterson DR, Carrougher GJ, Furness TA (2000). Virtual reality as an adjunctive pain control during burn wound care in adolescent patients. Pain.

[8] Hoffman HG, Garcia-Palacios A, Patterson DR, Jensen M, Furness T, Ammons WF (2001). The effectiveness of virtual reality for dental pain control: a case study. Cyberpsychol Behav.

[9] Morris LD, Louw QA, Crous LC (2010). Feasibility and potential effect of a low-cost virtual reality system on reducing pain and anxiety in adult burn injury patients during physiotherapy in a developing country. Burns.

[10] Dascal J, Reid M, IsHak WW, Spiegel B, Recacho J, Rosen B, Danovitch I (2017). Virtual reality and medical inpatients: a systematic review of randomized, controlled trials. Innov Clin Neurosci.

[11] Mosadeghi S, Reid MW, Martinez B, Rosen BT, Spiegel BMR (2016). Feasibility of an immersive virtual reality intervention for hospitalized patients: an observational cohort study. JMIR Ment Health.

[12] Schuster-Amft C, Eng K, Lehmann I, Schmid L, Kobashi N, Thaler I, Verra ML, Henneke A, Signer S, McCaskey M, Kiper D (2014). Using mixed methods to evaluate efficacy and user expectations of a virtual reality-based training system for upper-limb recovery in patients after stroke: a study protocol for a randomised controlled trial. Trials.

[13] Akhtar K, Sugand K, Wijendra A, Standfield NJ, Cobb JP, Gupte CM (2015). Training safer surgeons: How do patients view the role of simulation in orthopaedic training?. Patient Saf Surg.

[14] Lewis GN, Woods C, Rosie JA, McPherson KM (2011). Virtual reality games for rehabilitation of people with stroke: perspectives from the users. Disabil Rehabil Assist Technol.

[15] Thornton M, Marshall S, McComas J, Finestone H, McCormick A, Sveistrup H (2005). Benefits of activity and virtual reality based balance exercise programmes for adults with traumatic brain injury: perceptions of participants and their caregivers. Brain Inj.

[16] Constantine J (2017). TechCrunch, Oath Tech Network.

[17] (2016). Facebook.

[18] McCambridge J, Witton J, Elbourne DR (2014). Systematic review of the Hawthorne effect: new concepts are needed to study research participation effects. J Clin Epidemiol.

[19] Bowler GM (2010). Netnography: a method specifically designed to study cultures and communities online. Qual Rep.

[20] McEvenue G, Copeland A, Devon KM, Semple JL (2016). How social are we? a cross-sectional study of the website presence and social media activity of Canadian plastic surgeons. Aesthet Surg J.

[21] Benetoli A, Chen TF, Schaefer M, Chaar BB, Aslani P (2016). Professional use of social media by pharmacists: a qualitative study. J Med Internet Res.

[22] AlQarni ZA, Yunus F, Househ MS (2016). Health information sharing on facebook: an exploratory study on diabetes mellitus. J Infect Public Health.

[23] Kent EE, Prestin A, Gaysynsky A, Galica K, Rinker R, Graff K, Chou WS (2015). “Obesity is the new major cause of cancer”: connections between obesity and cancer on facebook and twitter. J Cancer Educ.

[24] Schwartz HA, Eichstaedt JC, Kern ML, Dziurzynski L, Ramones SM, Agrawal M, Shah A, Kosinski M, Stillwell D, Seligman ME, Ungar LH (2013). Personality, gender, and age in the language of social media: the open-vocabulary approach. PLoS One.

[25] Park G, Yaden DB, Schwartz HA, Kern ML, Eichstaedt JC, Kosinski M, Stillwell D, Ungar LH, Seligman ME (2016). Women are warmer but no less assertive than men: gender and language on facebook. PLoS One.

[26] Venkatesh V, Morris MG, Ackerman PL (2000). A longitudinal field investigation of gender differences in individual technology adoption decision-making processes. Organ Behav Hum Decis Process.

[27] Venkatesh V, Morris M (2000). Why don't men ever stop to ask for directions? Gender, social influence, and their role in technology acceptance and usage behavior. MIS quarterly.

[28] Riquelme H, Rios R (2010). The moderating effect of gender in the adoption of mobile banking. Intl Jnl of Bank Marketing.

[29] Weber RF (1990). Basic Content Analysis. Sage University Paper series on Quantitative Applications in the Social Sciences, 2nd Edition.

[30] Thelwall M, Wilkinson D, Uppal S (2009). Data mining emotion in social network communication: gender differences in MySpace. J Am Soc Inf Sci.

[31] Sullivan C, Schneider PE, Musselman RJ, Dummett CO, Gardiner D (2000). The effect of virtual reality during dental treatment on child anxiety and behavior. ASDC J Dent Child.

[32] Gershon J, Zimand E, Pickering M, Rothbaum BO, Hodges L (2004). A pilot and feasibility study of virtual reality as a distraction for children with cancer. J Am Acad Child Adolesc Psychiatry.

[33] Katz JN, Fossel KK, Simmons BP, Swartz RA, Fossel AH, Koris MJ (1995). Symptoms, functional status, and neuromuscular impairment following carpal tunnel release. J Hand Surg Am.

[34] Lewis GN, Rosie JA (2012). Virtual reality games for movement rehabilitation in neurological conditions: how do we meet the needs and expectations of the users?. Disabil Rehabil.

[35] Farrow S, Reid D (2004). Stroke survivors' perceptions of a leisure-based virtual reality program. Technol and Disabil.

[36] Kramer TL, Pyne JM, Kimbrell TA, Savary PE, Smith JL, Jegley SM (2010). Clinician perceptions of virtual reality to assess and treat returning veterans. Psychiatr Serv.

[37] Thomas JS, France CR, Applegate ME, Leitkam ST, Walkowski S (2016). Feasibility and safety of a virtual reality dodgeball intervention for chronic low back pain: a randomized clinical trial. J Pain.

[38] Liao Y, Yang Y, Cheng S, Wu Y, Fuh J, Wang R (2015). Virtual reality-based training to improve obstacle-crossing performance and dynamic balance in patients with Parkinson's disease. Neurorehabil Neural Repair.

[39] Matar E, Shine JM, Naismith SL, Lewis SJ (2013). Using virtual reality to explore the role of conflict resolution and environmental salience in freezing of gait in Parkinson's disease. Parkinsonism Relat Disord.

[40] Robles-García V, Corral-Bergantiños Y, Espinosa N, García-Sancho C, Sanmartín G, Flores J, Cudeiro J, Arias P (2016). Effects of movement imitation training in Parkinson's disease: a virtual reality pilot study. Parkinsonism Relat Disord.

[41] Bordnick PS, Traylor AC, Carter BL, Graap KM (2012). A feasibility study of virtual reality-based coping skills training for nicotine dependence. Res Soc Work Pract.

[42] Gonçalves R, Pedrozo AL, Coutinho ES, Figueira I, Ventura P (2012). Efficacy of virtual reality exposure therapy in the treatment of PTSD: a systematic review. PLoS One.

[43] Pietrzak E, Pullman S, McGuire A (2014). Using virtual reality and videogames for traumatic brain injury rehabilitation: a structured literature review. Games Health J.

[44] Funk C, Kennedy B, Podrebarac Sciupac E (2016). Pew Research Center.

[45] Anderson M (2015). Pew Research Center.

[46] Lenhart A, Purcell K, Smith A, Zickuhr K (2010). Pew Research Center.

[47] Shih P, Han K, Poole E, Rosson M, Carroll J (2015). ResearchGate.

[48] Nysveen H, Pedersen P, Thorbjørnsen H (2005). Explaining intention to use mobile chat services: moderating effects of gender. J Consum Mark.

[49] Lucas K, Sherry J (2016). Sex differences in video game play: a communication-based explanation. ‎Commun Res.

[50] Weiser E (2000). Gender differences in internet use patterns and internet application preferences: a two-sample comparison. Cyberpsychol Behav.

