# Supplementary figures and images for: Public Perceptions Regarding Use of Virtual Reality in Health Care: A Social Media Content Analysis Using Facebook
Source: J Med Internet Res. 2017 Dec 19;19(12):e419. doi: 10.2196/jmir.7467 (PMC5750416; doi:10.2196/jmir.7467)

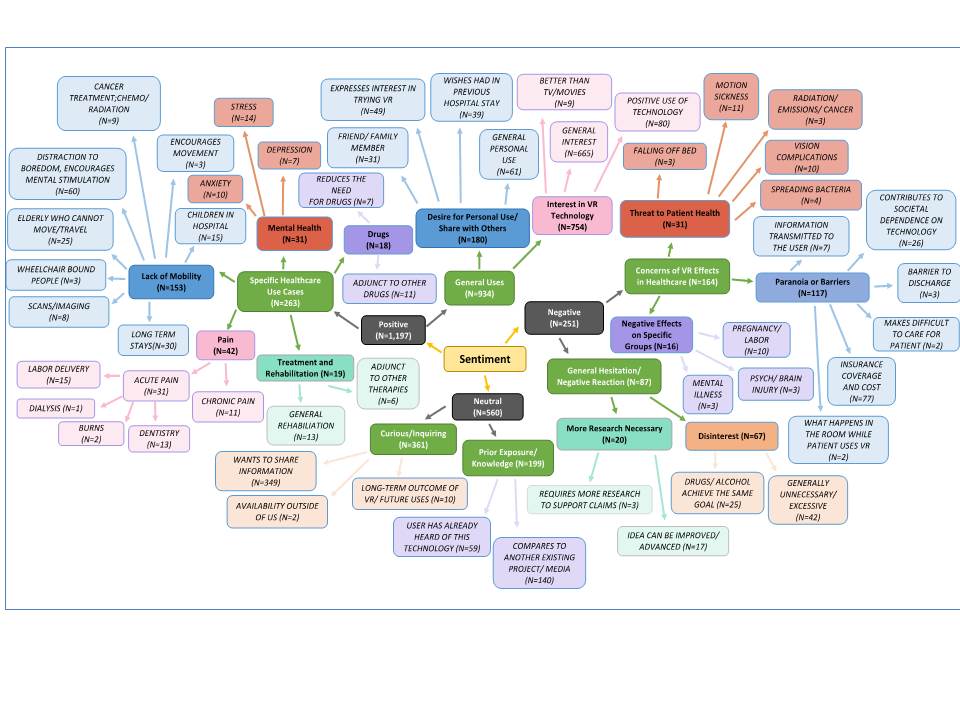

Supplement: Multimedia Appendix 1 [file jmir_v19i12e419_app1.jpg]
