# Supplementary material for: Public Perceptions Regarding Use of Virtual Reality in Health Care: A Social Media Content Analysis Using Facebook
Source: J Med Internet Res. 2017 Dec 19;19(12):e419. doi: 10.2196/jmir.7467 (PMC5750416; doi:10.2196/jmir.7467)
Supplement: Multimedia Appendix 2 [file jmir_v19i12e419_app2.pdf]

Multimedia Appendix 2. Examples of Facebook Comments about VR Technology in the Healthcare Setting

	Elderly Patients Who Cannot Move or Travel	Specify Setting	Stress	Anxiety/Depression	Adjunct to Drugs	Positive Use of Technology	Wishes had VR for Friend or Family Member	Insurance Coverage and Cost
"I can see the benefits, but having a lengthy hospital stay myself, in the past, I can only say that they should make the environment more aesthetically pleasing, with colours, artist work, softer lighting, and quieter. Hospitals are not healing places. The staff also is not present most of the time to the patients needs, they are overworked and understaffed most of the time, or just don't care enough. I found the environment as hard to take as the illness itself."								
"Anyone who has seen a family member lose their faculties and become disoriented, because of a variety of reasons including lack of sleep in a hospital setting, can appreciate the value of this technology. Just the mental stimulation would help the bed ridden patient so much."	✓	✓				✓	✓	
"I'm sure this tech could make stays in hospital more bearable and even improve patient recovery and result in shorter patient stays. But the short sighted buffoons that run hospitals will view these as a profit centre and like internet and phone access from hospital it will be priced so that no one, except the very wealthy can afford it (when Jan was in hospital a few years ago I looked at the internet prices and concluded that it wasn't even value for money for someone like me that could use it to moniter their business from hospital - the pricing is that bad)."			✓			✓		✓
"This would be good for mentally ill patients in hospitals to be used as a medical tool for bringing patients into reality when they have been through trama in their life and gone out of their mind, confused, disoriented, between past and present and staff do not read medical records and also mix past and present up, who is mentally ill? Maybe cognitive therapy or Christian minfulness can be done using this technology. Also patients on dialysis as are on machine for at least 3-4 hrs could benefit from this technology. will NHS with all the cuts bother at all."		✓		✓	✓			
